# Evaluation of Thyroid Functions with Respect to Iodine Status and TRH Test in Chronic Autoimmune Thyroiditis

**DOI:** 10.4274/jcrpe.v3i1.04

**Published:** 2011-02-23

**Authors:** Ayça Törel Ergür, Olcay Evliyaoğlu, Zeynep Şıklar, Pelin Bilir, Gönül Öcal, Merih Berberoğlu

**Affiliations:** 1 Ufuk University School of Medicine, Department of Pediatric Endocrinology, Ankara, Turkey; 2 Ankara University School of Medicine, Department of Pediatric Endocrinology, Ankara, Turkey; +90 312 266 67 78aycaergur@superonline.comUfuk University School of Medicine, Department of Pediatric Endocrinology, Ankara, Turkey

**Keywords:** Chronic autoimmune thyroiditis, childhood, adolescent

## Abstract

**Objective:** Chronic autoimmune thyroiditis (CAT) is the most common form of thyroiditis in childhood and a frequent cause of acquired hypothyroidism. The objective of this study was to evaluate  the thyroid status of childrenand adolescents with CAT with respect to iodine status and diagnostic values of thyrotropin-releasing hormone (TRH) test.

**Methods:** Seventy-one children (mean age: 11.6 years) were studied in a retrospective analysis. Free thyroxine (T4), thyrotropin (TSH), TSH response to TRH test, thyroid autoantibodies, thyroid sonography, and urinary iodine excretion (UIE) were evaluated.

**Results:** At diagnosis, 8.5% of patients had overt hypothyroidisim and 36.6% subclinical hypothyroidism; 5.6% had overt hyperthyroidisim and 8.5% had subclinical hyperthyroidism. Of them, 40.8% were euthyroid. Median UIE was 51 mg/L in overt hypothyroidism and 84 mg/L in subclinical hypothyroidism. The values were 316 mg/L and 221 mg/L in overt and subclinical hyperthyroidism, respectively. Basal TSH showed a strong correlation with peak TSH level on TRH test. Thirty-four percent of patients with normal basal TSH level showed an exaggerated TSH response.

**Conclusion:** Iodine deficiency was seen more in cases with hypothyroidism, while excess of iodine was observed to be more frequent in hyperthyroid patients. Iodine status was a strong predictorof the thyroid status in CAT. TRH test may be helpful in further delineating patients with subclinical hypothyroidism.

**Conflict of interest:**None declared.

## INTRODUCTION

Primary acquired childhood and juvenile hypothyroidism is mainly due to chronic autoimmune thyroiditis (CAT) (1). Because of the risk of hypothyroidism, the disease requires lifelong thyroid surveillance, especially in women of child-bearing age in order to avoid adverse effects on the expected child. The aim of this study was to evaluate the clinical and laboratory features of CAT in childhood and adolescence in our patient population mainly with respect to iodine levels and thyroid status as well as to diagnostic values of thyrotropin-releasing hormone (TRH) test.

## METHODS

Seventy-one children with a mean age of 11.6±2.7 (range: 5.4-17.5) years were studied in a retrospective analysis. All cases were evaluated with a detailed physical examination, pubertal staging (according to Tanner staging), and height and weight measurements. Height was expressed as standard deviation score (SDS) (2). Body mass index (BMI) was calculated as: weight (kg)/height (m)^2^. Cases with a height below -2 SDS were considered to have a short stature and those with BMI above >95^th^ were considered obese (2,3). Relative BMI was calculated by dividing the individual's BMI to normal BMI adjusted for sex and age (100% x individual's BMI/sex- and age-specific BMI cut-offs from a reference population) (3).

The thyroid gland was assessed by palpation and graded according to the goitre classification system proposed by the World Health Organization (WHO) (4). Serum free triiodothyronine (T3), free thyroxine (T4), thyrotropin (TSH), antithyroglobulin (anti-TG), antithyroid peroxidase antibody (anti-TPO) were measured in all patients. Serum free T3, free T4 levels were determined by competitive immunoassay method using immunodiagnostic products (5). Serum TSH levels were measured by immunometric radioimmunoassay (IRMA) method. Anti-TG and anti-TPO were measured using immunometric assay method (Immulite 2000®, DPC, Los Angeles) .Values above 35 U/ml for anti-TG and above 40 U/ml for anti-TPO were considered positive (6). Standard TRH test (7) ( giving TRH in a dose of 5-7 mg/kg body weight and measuring TSH at 0, 20, 40 and 60 minutes) was performed  in all patients without overt hypothyroidism or overt hyperthyroidism. Overt hypothyroidism was accepted in the presence of high basal TSH level with low T4 level. Patients with subclinical hypothyroidism had normal T4 and elevated basal TSH. The diagnosis of hyperthyroidism was established in the presence of suppressed basal TSH level (TSH below 0.5 mIU/ml) with high (overt hyperthyroidism) or normal (subclinic hyperthyroidism) T4 level. TSH response to TRH was considered normal if the peak TSH level was between 5-25 mIU/L. A peak value above 25 mIU/L was considered as exaggerated and a value below 5 mIU/L as suppressed (7).

Thyroid sonography was performed by high-resolution ultrasound using 7.5 MHz probes in each patient. Thyroid volumes were calculated by the Neu’s reference criteria (8). Longitudinal and transverse scans were performed allowing the measurement of the depth, length and width of each lobe. Thyroid volume was taken as the sum of the volumes of the two lobes. The volume of the isthmus was not included. Accordingly, cases with thyroid volumes above 97^th^ percentile were accepted to have goitre (9). The diagnosis of CAT on ultrasonographic evaluations  included varying degree of hypoechogenicity, heterogeneous parenchyma, peppered with innumerable small hypoechoic nodules measuring a few millimeters and separated by echogenic septae (10).

Morning urinary samples were taken from all patients for determination of urinary iodine excretion (UIE) which was measured by colorimetric method suggested by WHO-ICCIDD (11). The prevalence of iodine deficiency was graded according to the WHO classification (12). UIE levels between 100 and 200 μg/L were accepted as normal. Mild, moderate or  severe iodine deficiency was present when UIE was 50-99 μg/L, 20-49 μg/L, or <20 μg/L, respectively. A value greater than 200 μg/L indicated excessive urinary iodine.

LT4 treatment was initiated in cases whose thyroid gland was over +2 SDS and in cases with overt or subclinical hypothyroidism or with normal basal TSH levels but with exaggerated TSH response on TRH test. Statistical analysis was performed using the Statistical Package for the Social Sciences (SPSS) version 10.0 for Windows. The values for continuous variables have been shown as the mean (SDS) and median. 

## RESULTS

The mean age of the patients at diagnosis was 11.6±2.7 years, height SDS was -0.3±0.8 SDS, BMI was 95.7±14.4%. Twenty-two out of the seventy-one patients (30%) were prepubertal. There was a female preponderance (n=61, 85.9%).  All children had palpable goitre. The goitre stages were 1a in 13%, 1b in 28%, II in 42%, and III in 16.9%. Two cases had short stature with hypothyroidism. Obesity was encountered in six patients (8.5%), four of whom had also subclinical hypothyroidism. Twenty percent had a family history of thyroid disease. 

The severity of iodine deficiency according to the median UIE in all patients is shown in Table 1. Iodine deficiency was determined in 59.1% of the total group. Excessive urinary iodine concentration was observed in 12.7%.  

**Figure 1:**
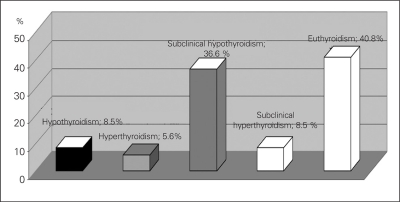
Thyroid status in the CAT patients CAT: chronic autoimmune thyroiditis

As for the thyroid functions at diagnosis, 6 patients (8.5%) had overt hypothyroidism with high TSH levels, nine (36.6%) had borderline increased levels of TSH (between 5 to 10 mIU/ml) and normal fT4 levels, and 38 children (40.8%) had normal basal thyroid hormone and TSH levels. Ten patients had suppressed TSH levels, with normal thyroid hormone levels in 6 (8.5%) (subclinical hyperthyroidisim) and with high thyroid hormone levels in 4 (5.6%) (overt hyperthyroidism) (Figure 1). TRH stimulation test was applied to 61 patients without overt hypothyroidism or overt hyperthyroidism. Stimulated TSH response positively correlated with basal TSH levels (r=0.88, p<0.000). UIE negatively correlated with stimulated TSH levels (r=-0.35, p=0.014). The response to TRH test according to basal TSH level and UIE are shown in Table 2. Patients (34%) with normal basal TSH levels had an exaggerated TSH response on TRH test. Moreover, of those with basal TSH levels between 2 and 5 mIU/mL, 50% had an exaggerated TSH response. According to thyroid dysfunction types, the median UIE values are shown in Table 3. Iodine deficiency was prevalent in CAT patients who had overt or subclinical hypothyroidism and iodine excess in hyperthyroidism.

Anti-TG antibodies were positive in 80.3% (n=58) and anti-TPO antibodies in 71.8% (n=51) of subjects. Both antibodies were positive in 53.5% patients (n=38). TSH receptor antibodies (TRAb) were negative in all patients. 

On thyroid USG, a heterogeneous parenchymal structure was observed in 58% of  patients and nodules were noted in 7% of cases. 

Patients on LT4 treatment have been followed up for 3.26±2.8 years.  The LT4 therapy was not given to 10 cases at admission, but was started after a follow-up of 0.9±0.6 years because hypothyroidism developed. Hyperthyroidism did not persist in any of the subclinical or overt hyperthyroid patients. 

**Table 1:**
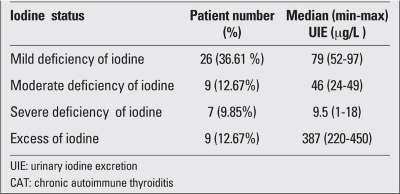
The iodine status in the CAT patients

**Table 2:**
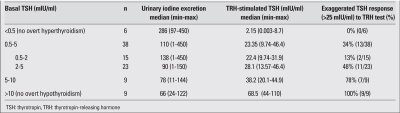
TSH response to TRH test according to basal TSH

**Table 3:**
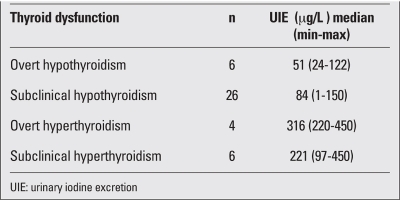
Iodine status in different thyroid dysfunction types

## DISCUSSION

CAT  is one of very frequently seen thyroid pathologies which may lead to thyroid dysfunction, particularly hypothyroidism (13). 

The general clinical features of the patients and the percentage of family history in our study are consistent with the published in the literature (14). However, we observed a relatively higher rate of thyroid dysfunction than cited in the literature (15), which may be due to the concomitant presence of iodine deficiency in our series.

Indeed, in another study conducted in our country, 24.1% of CAT patients (n=162) had compensated hypothyroidism, 21% had overt hypothyroidism, 8.6%-overt hyperthyroidism, and 3.1% had subclinical hyperthyroidism (16). The rate of thyroid dysfunction in CAT may be high in Turkey (17).

In our study, CAT was accompanied by iodine deficiency in 50% of cases. While iodine deficiency was seen more in cases of hypothyroidism, excess of iodine was observed to be more frequent in hyperthyroid patients. Iodine deficiency seems to increase the risk of hypothyroidism. Euthyroid patients had almost normal UIE. In CAT patients showing either overt or subclinical hypothyroidism, iodine excess was frequently seen instead of iodine deficiency. It is well known that iodine-induced hyperthyroidism may occur in patients with iodine-deficiency goitre, in euthyroid Graves’ disease patients after antithyroid drug therapy, in euthyroid subjects with previous spontaneous and iatrogenic episodes of thyroid dysfunction, in patients with multinodular goitres who reside in areas of iodine repletion or deficiency (18). Hyperthyroidism was transient in our CAT patients, and none of them had positive TRAb; therefore, there was no coexistence of CAT and Graves’ diseases.  

In the presence of autoimmune  thyroid disease, the control of UIE may be  helpful in predicting probable thyroid dysfunction and may lead our recommendation on the use of iodized salt. Some researchers suggest that increase in iodine intake triggers autoimmunity and may increase CAT prevalence (19). However, our findings do not verify this observation since a substantial number of children had iodine deficiency. The rate of positive antibodies in our study is relatively similar to the rates cited in the literature (15,16).

As for LT4 therapy during childhood in CAT patients with subclinical hypothyroidism, no controlled studies have been reported in the literature. Assuming that development may be affected adversely, LT4 treatment was initiated in such cases in this study. We believe that the follow-up evaluation of these children will  have a significant role fromthis standpoint. Another important finding is that basal TSH showed a very strong association with peak TSH level on TRH test and this may lead to the conclusion that a TRH test is not needed to assess the thyroid status. Indeed, in those with suppressed TSH or increased basal TSH levels, TRH test does not seem to further add to the diagnosis. However, nearly one third of our patients with normal TSH levels had an exaggerated TSH response on TRH test. Especially those with a TSH level between 2 and 5mIU/mL had frequently an exaggerated TSH response. Future long-term studies and follow-up of these children are needed to explore whether this implies an abnormality.

In conclusion, iodine status has an effect on the thyroid functions in CAT. TRH test may help identifying patients with subclinical hypothyroidism. 
